# Psychological factors, lifestyle habits, and their association with gastroesophageal reflux disease among Egyptian university students: A cross-sectional study

**DOI:** 10.1097/MD.0000000000040477

**Published:** 2024-11-22

**Authors:** Hebatalla Abdelmaksoud Abdelmonsef Ahmed, Ahmed Yousef, Rania El-Kurdy, Manal Abdulaziz Murad, Shady Mohamed Abdelwahab, Hoda Ali Ahmed Shiba

**Affiliations:** a Public Health & Community Medicine, Faculty of Medicine, Kafr-Elsheikh University, Kafr El Sheikh, Egypt; b Public Health and Community Medicine, Damietta Faculty of Medicine, Al-Azhar University, Cairo, Egypt; c Woman’s Health and Midwifery Nursing, Faculty of Nursing, Mansoura University, Mansoura, Egypt; d Family Medicine, Family and Community Medicine Department, Faculty of Medicine, Rabigh, King Abdulaziz University, Jeddah, Saudi Arabia; e Faculty of Medicine, Kafr-Elsheikh University, Kafr El Sheikh, Egypt; f Public Health & Community Medicine, Faculty of Medicine, Al-Azhar University, Cairo, Egypt.

**Keywords:** anxiety, dietary habits, perceived stress, physical activity, university students

## Abstract

Gastroesophageal reflux disease (GERD) is a common condition among university students that is associated with various lifestyle and psychological factors. The study aims to evaluate the association of psychological factors, physical activity, and dietary habits with the prevalence of GERD among university students in Egypt. An analytical cross-sectional study was conducted from March 2024 to May 2024, selecting students from different universities in Egypt. A self-administered questionnaire covered sociodemographic characteristics, and GERD assessment using the Gerd Q questionnaire. Generalized Anxiety Disorder-2, Perceived Stress Scale 4, Nordic Physical Activity Questionnaire, and short dietary habits questions were used to assess anxiety, perceived stress, physical activity, and dietary habits, respectively. Statistical analysis included descriptive statistics, correlation analysis, and hierarchical logistic regression, which were employed to identify significant predictors of GERD. Among the 2241 university students, 22.6% had GERD (GerdQ score > 8), 86.3% had high perceived stress, and 62.2% had high anxiety levels. Hierarchical logistic regression analysis identified old age, high weight, short height, frequent consumption of fried/fast food, and high anxiety levels as significant predictors of GERD. The study underscores the importance of considering psychological factors and dietary habits alongside traditional risk factors in understanding and addressing GERD among university students in Egypt. Public health interventions targeting psychological well-being and healthier dietary habits will play a pivotal role in reducing this population’s GERD burden.

## 1. Introduction

Gastroesophageal reflux disease (GERD) is a chronic condition that affects the digestive system’s upper tract and can impact a patient’s health-related quality of life.^[1]^ It has emerged as a major global public health challenge today, affecting millions and putting a strain on healthcare systems worldwide.^[2]^ Up to 20% of the population globally have GERD. An increasing prevalence of GERD could have some reflection on the fact that life expectancy has now increased worldwide.^[3]^ GERD poses a substantial health and economic burden because of its widespread incidence, impact on daily life, young age of the individual, and possibility for future complications.^[4]^ The interactions of environmental factors, genetic predisposition, and changes in food and physical activity have all contributed to its prevalence.^[5]^

Numerous lifestyle habits, including high-fat, spicy, or acidic foods, can trigger GERD symptoms. Fast food, which is common among young people, is particularly problematic. Eating habits, such as late-night eating or skipping meals, also play a role.^[6]^ Poor sleeping habits might exacerbate GERD symptoms.^[7]^ These habits are more common in adolescence and young adulthood, which contributes to the prevalence of GERD in this age group.^[8]^ Traditional Egyptian diets may contain spicy meals, high-fat content, and beverages such as tea or coffee, all of which can exacerbate GERD symptoms.^[9]^

Additionally, academic pressures, social interactions, and life transitions can all cause stress in university students, increasing their susceptibility to GERD.^[10]^ Stress is a significant factor that might worsen GERD symptoms. High levels of stress or anxiety can boost stomach acid production, resulting in more severe reflux symptoms.^[11]^ Depression is also associated with GERD, probably because of changes in eating patterns, sleep difficulties, and the overall influence on physical health.^[12]^ Several studies have demonstrated the vicious cycle between GERD and psychological disorders; patients with GERD are more prone to acquire psychosocial disorders than healthy persons; nevertheless, psychosocial disorders can also increase the risk of GERD.^[13–15]^

Studies in Western contexts have established the association between different psychological disorders and the exacerbation of GERD symptoms. These studies noted that psychological distress can lead to increased acid production and altered esophageal function.^[15]^ Egyptian university students face unique psychosocial stressors, including academic stress, financial hardship, low resilience, low socioeconomic status, and broader cultural and societal factors.^[16]^ These stressors can contribute to the development and exacerbation of GERD. Understanding the Egyptian context of these factors is crucial for identifying tailored interventions that can help reduce the burden of GERD by exploring how these psychosocial stressors intersect with lifestyle habits to influence the condition among Egyptian university students. To the best of our knowledge, limited research to date has investigated the combined effects of psychological stress, lifestyle factors, and GERD in Egyptian university students. Therefore, the objectives of the current study are to evaluate the association of psychological factors, physical activity, and dietary habits with the prevalence of GERD among university students in Egypt. By focusing on this population, we will provide insights into the prevalence and drivers of GERD, as well as the potential for targeted interventions to mitigate its impact.

## 2. Methods

### 2.1. Study design and population

An analytical cross-sectional study was conducted from March 2024 to May 2024 on undergraduate Egyptian university students who accepted to participate. We employed a stratified sampling method to ensure a comprehensive and representative sample of university students across Egypt. Egypt is administratively grouped into 4 distinct geographical areas: Urban Governorates, the Nile Delta (Lower Egypt), the Nile Valley (Upper Egypt), and the Frontier Governorates. We sampled students from Cairo University in Cairo, representing the Urban Governorates. In the Nile Delta, we sampled students from Kafr-Elsheikh University in Kafr-Elsheikh. For the Nile Valley, we sampled students from Assiut University in Assiut. To include students’ perspectives from Egypt’s frontier regions, we selected Arish University in North Sinai. We selected specific universities based on our established academic contacts, research assistants, and student representatives from each university. This sampling strategy provides a balanced and representative view of university students across Egypt’s diverse regions, ensuring that the study’s findings are generalizable to the broader population.

### 2.2. Sample size and technique

The sample size was determined using the Epi-info software statistical package.^[17]^ The criteria considered included a 95% confidence level, 80% power, a margin of error of 2.5%, and an expected GERD prevalence of 17.1% among university students in Egypt from a prior study.^[18]^ Accordingly, the minimum required sample size was calculated to be 1360 students. To account for a nonresponse rate and incomplete or inconsistent data the final sample size was adjusted to 2241 students. The participants were selected using a multistage stratified sampling approach. At first, we divided the faculties in each selected university into 2 groups based on the specialty (medical and non-medical). Then, we randomly selected 1 faculty from each group. After this, we retrieved lists of all students enrolled in each university’s 2 selected faculties to randomly and proportionately select students from different academic years.

### 2.3. Data collection methods

An anonymous self-administered questionnaire was used to investigate the prevalence and influential factors of GERD among university students in Egypt. To ensure its validity, 6 professors specializing in community medicine, psychiatry, and internal medicine juried the questionnaire. Additionally, the questionnaire’s reliability was assessed by examining the internal consistency of each domain through Cronbach Alpha.

### 2.4. The questionnaire included 5 parts

(1) Sociodemographic and general characteristics of the studied participants, including information about age, sex, residence, university, and faculty. In addition, self-reported height and weight were inquired, then body mass index (BMI) was calculated.
(2) GerdQ: The GerdQ questionnaire is a validated tool designed to assess the probability of GERD. It comprises 4 positive queries aimed at evaluating GERD symptoms like heartburn, regurgitation, sleep disturbances induced by heartburn and regurgitation, and medication usage, alongside 2 negative inquiries regarding epigastric pain and nausea. Scoring in GerdQ is based on the frequency of these symptoms experienced in the preceding week, ranging from less than once to 4 to 7 times, with scores varying from 0 to 3 for positive GERD indicators and in the opposite order for negative ones (where 3 indicates absence of symptoms). Individuals scoring above 8 on the GerdQ scale were identified as having GERD. The Arabic version of GerdQ was specifically developed and validated for Arabic-speaking populations. Internal consistency was evaluated in the present study using Cronbach Alpha, yielding a coefficient of 0.685.
(3) Generalized Anxiety Disorder-2 (GAD-2): The GAD-2 serves as a psychometrically valid screening instrument for anxiety symptoms assessment, consisting of the initial 2 questions of the GAD-7: “feeling nervous, anxious, or on edge” and “not being able to stop or control worrying..”^[19]^ Each item is rated on a scale from 0 (not at all) to 3 (nearly every day), leading to a cumulative score ranging from 0 to 6. A score of 3 or higher is the suggested threshold for identifying individuals who may necessitate further evaluation for generalized anxiety disorder. In the current study, internal consistency was assessed using Cronbach Alpha, yielding a coefficient of 0.811.
(4) Perceived Stress Scale 4 (PSS-4): PSS-4 is a concise self-report measure designed to evaluate psychological stress levels.^[20]^ It comprises 4 items, each rated on a 5-point Likert scale from 0 (“never”) to 4 (“very often”), with 2 items reverse-coded. Scores range from 0 to 16, with higher scores indicating higher perceived stress. A PSS-4 score of 6 was utilized to classify individuals experiencing elevated levels of self-perceived stress. Internal consistency was assessed using Cronbach Alpha, resulting in a coefficient of 0.677.
(5) Nordic Physical Activity Questionnaire (NPAQ) short: The NPAQ short is a survey instrument that gauges physical activity through 2 questions. One question pertains to moderate to vigorous physical activity (MVPA), while the other addresses vigorous physical activity (VPA). These questions take into account the duration of physical activity over the past week to align with the World Health Organization’s recommendations on physical activity for populations. Internal consistency was measured using Cronbach Alpha (α = 0.758).
(6) Dietary habits questions: The dietary habit was assessed through 2 items about the frequency of eating fried/fast food and the frequency of eating fruits and vegetables. The items were assessed on the following scale: Never = 0, rarely (one per week) = 1, sometimes (more than 2 per week) = 2, and usually (daily) = 3.

Selecting the shorter versions of psychometric scales, specifically the GAD-2 and the PSS-4, in our study was primarily driven by practical considerations regarding respondent burden and study efficiency. University students often face time constraints and academic pressures, making shorter instruments more feasible for completion. Both the GAD-2 and PSS-4 have demonstrated robust psychometric properties including high reliability and validity in various populations.^[21,22]^

### 2.5. A pilot study

It was conducted with a cohort of 50 university students to evaluate the study tools concerning their clarity, feasibility, applicability, and the time needed to complete the questionnaires. Accordingly, some adjustments were implemented.

### 2.6. Data management and analysis

The Statistical Package for the Social Sciences “SPSS” 22.0 software (IBM Microsoft) was used to analyze data. Incomplete and inconsistent participant data were excluded from the analysis to ensure data quality. Numerical data normality was explored using Kolmogorov test. Numbers and percentages presented categorical variables, and for analysis, the Chi-square test was used. Numerical variables were presented as means and standard deviations, and the Mann–Whitney *U* test was used to compare groups. Spearman correlation analysis evaluated the relationship between the studied variables. A hierarchical logistic regression analysis was employed to assess the relationship between various predictors (have *P*-value ≤ .2 in bivariate analysis) and GERD. In the first step of the model, we included sociodemographic variables such as age, medical specialty, weight, height, and BMI, which were identified as potential confounders. In the second step, lifestyle factors, including dietary habits, were added to the model. In the final step, psychological factors, including anxiety (GAD-2) and perceived stress (PSS-4), were introduced to assess their independent association with GERD. *P*-value (<.05) was adopted as the level of significance.

## 3. Results

### 3.1. The study participants’ socio-demographic and personal characteristics based on their GERD diagnosis

Among 2241 University students, gender distribution showed a higher representation of females (70.1%). University participation displayed diversity, with Kafr-Elsheikh University contributing nearly half of the participants (48.3%). The division between medical (26.3%) and non-medical (73.3%) specialties appears skewed towards non-medical fields. Additionally, more than half (56.4%) fell within the normal weight range. Regarding GERD, 22.6% of the study participants were diagnosed with GERD (GerdQ score > 8). Sex, residence, universities, and specialty showed statistically insignificant differences based on GERD diagnosis. However, the mean age, weight, and BMI were significantly higher among students with GERD than those without. In addition, the distribution of participants across body weight categories showed a significant difference in GERD prevalence (Table 1).

**Table 1:** Socio-demographic and personal characteristics of study participants based on their GERD (gastroesophageal reflux disease) diagnosis.

Studied variables		Total	GERD status		
			Yes (GerdQ > 8)	No (GerdQ ≤ 8)	Total GerdQ scores
		N (%)	N (%)	N (%)	Mean ± SD
Overall		2241 (100%)	507 (22.6%)	1734 (77.4%)	7.1 ± 2.1
Gender	Male	669 (29.9%)	142 (21.2%)	527 (78.8%)	7.1 ± 2.0
	Female	1572 (70.1%)	365 (23.2%)	1207 (76.8%)	7.1 ± 2.2
	*P*-value		.302		.777
Age (years)	Mean ± SD	20.2 ± 1.6	20.4 ± 1.7	20.2 ± 1.5	–
	*P*-value		.031*		–
Residence area	Urban area	1210 (54%)	275 (22.7%)	935 (77.3%)	7.1 ± 2.2
	Rural area	1031 (46%)	232 (22.5%)	799 (77.5%)	7.1 ± 2.1
	*P*-value		.899		.656
University	Assiut University	344 (15.4%)	82 (23.8%)	262 (76.2%)	7.1 ± 2.3
	Arish University	389 (17.4%)	94 (24.2%)	295 (75.8%)	7.3 ± 2.2
	Cairo University	425 (19%)	96 (22.6%)	329 (77.4%)	6.9 ± 2.2
	Kafr-Elsheikh University	1083 (48.3%)	235 (21.7%)	848 (78.3%)	7.1 ± 2.1
	*P*-value		.718		.234
Specialty	Medical	590 (26.3%)	123 (20.8%)	467 (79.2%)	7.1 ± 1.9
	Non-medical	1651 (73.7%)	384 (23.3%)	1267 (76.7%)	7.1 ± 2.2
	*P*-value		.230		.858
Weight (kg)	Mean ± SD	68.0 ± 15.0	70.0 ± 15.0	68.0 ± 14.0	–
	*P*-value		.015*		–
Height (cm)	Mean ± SD	167.0 ± 10.0	166.0 ± 10.0	167.0 ± 10.0	–
	*P*-value		.151		–
Body mass index (BMI) (kg/m^2^)	Total average (Mean ± SD)	24.5 ± 4.7	25.15 ± 4.76	24.34 ± 4.72	–
	*P*-value		≤.001*		
	Underweight (<18.5 kg/m^2^)	111 (5.0%)	16 (3.2%)	95 (5.5%)	6.4 ± 1.9
	Normal weight (18.5–<25 kg/m^2^)	1265 (56.4%)	273 (53.8%)	992 (57.2%)	6.9 ± 2.1
	Overweight (25–<30 kg/m^2^)	635 (28.3%)	151 (29.8%)	484 (28.0%)	7.3 ± 2.0
	Obese (>30 kg/m^2^)	230 (10.3%)	67 (13.2%)	163 (9.3%)	7.6 ± 2.38
	*P*-value		.011*		≤.001*

GERD = gastroesophageal reflux disease.

* Significant (*P* < .05).

### 3.2. The association between stress, anxiety, physical activity, dietary habits, and GERD Status among the study participants

Concerning psychological factors, 86.3% of university students had high-stress levels as measured by the PSS-4, and 62.2% had high anxiety levels as measured by the GAD-2. The mean PSS-4 scores and GAD-2 were lower in individuals without GERD compared to those with GERD, with a statistically significant difference (*P* < .05). Regarding physical activity, 60.1% of university students engaged in MVPA levels for <30 minutes. The comparable figure for VPA was 66.4%. The distribution of MVPA and VPA across different durations did not show significant differences in GERD prevalence (*P* > .05). In terms of dietary habits, the frequency of eating fried food/fast food and fruits and vegetables showed significant differences in GERD prevalence. Participants who reported less frequent eating of fried food/fast food and those who consumed fruits and vegetables less frequently were more likely to have GERD (Table 2).

**Table 2:** Comparative analysis of GERD (gastroesophageal reflux disease) status by psychological and lifestyle factors.

Studied variables			Total	GERD status		*P*-value
				Yes (GerdQ > 8)	No (GerdQ ≤ 8)	
PSS-4	Total (Mean ± SD)		8.66 ± 2.89	8.95 ± 2.85	8.57 ± 2.9	.003*
	High-stress level N (%)		1935 (86.3%)	452 (89.2%)	1483 (85.5%)	.036*
	Low-stress level N (%)		306 (13.7%)	55 (10.8%)	251 (14.5%)	
GAD-2	Total (Mean ± SD)		3.39 ± 1.73	3.74 ± 1.68	3.29 ± 1.73	≤.001*
	High anxiety level N (%)		1394 (62.2%)	353 (69.6%)	1041 (60%)	≤.001*
	Low anxiety level N (%)		847 (37.8%)	154 (30.4%)	693 (40%)	
NPAQ-short	MVPA	<30 minutes N (%)	1346 (60.1%)	301 (59.4%)	1045 (60.3%)	.709
		30–90 minutes N (%)	521 (23.2%)	122 (24.1%)	399 (23%)	
		90–150 minutes N (%)	216 (9.6%)	51 (10.1%)	165 (9.5%)	
		150–300 N (%)	90 (4%)	22 (4.3%)	68 (3.9%)	
		>300 minutes N (%)	68 (3%)	11 (2.2%)	57 (3.3%)	
	VPA	<30 minutes N (%)	1489 (66.4%)	334 (65.9%)	1155 (66.6%)	.480
		30–60 minutes N (%)	327 (14.6%)	79 (15.6%)	248 (14.3%)	
		60–90 minutes N (%)	197 (8.8%)	45 (8.9%)	152 (8.8%)	
		90–150 N (%)	129 (5.8%)	33 (6.5%)	96 (5.5%)	
		>150 minutes N (%)	99 (4.4%)	16 (3.2%)	83 (4.8%)	
Dietary habits	Frequency of eating fried/fast food	Never N (%)	110 (4.9%)	27 (5.3%)	83 (4.8%)	.013*
		Rarely N (%)	619 (27.6%)	113 (22.3%)	506 (29.2%)	
		Sometimes N (%)	1055 (47.1%)	247 (48.7%)	808 (46.6%)	
		Usually N (%)	457 (20.4%)	120 (23.7%)	337 (19.4%)	
	Frequency of eating fruit and vegetables	Never N (%)	47 (2.1%)	16 (3.2%)	31 (1.8%)	.003*
		Rarely N (%)	931 (41.5%)	180 (35.5%)	751 (43.3%)	
		Sometimes N (%)	746 (33.3%)	173 (34.1%)	573 (33%)	
		Usually N (%)	517 (23.1%)	138 (27.2%)	379 (21.9%)	

GAD-2 = The Generalized Anxiety Disorder 2-item, GERD = gastroesophageal reflux disease, MVPA = moderate to vigorous physical activity, NPAQ-short = The Nordic Physical Activity Questionnaire-short, PSS-4 = Perceived Stress Scale 4, VPA = vigorous physical activity.

* Significant.

**Figure 1.**
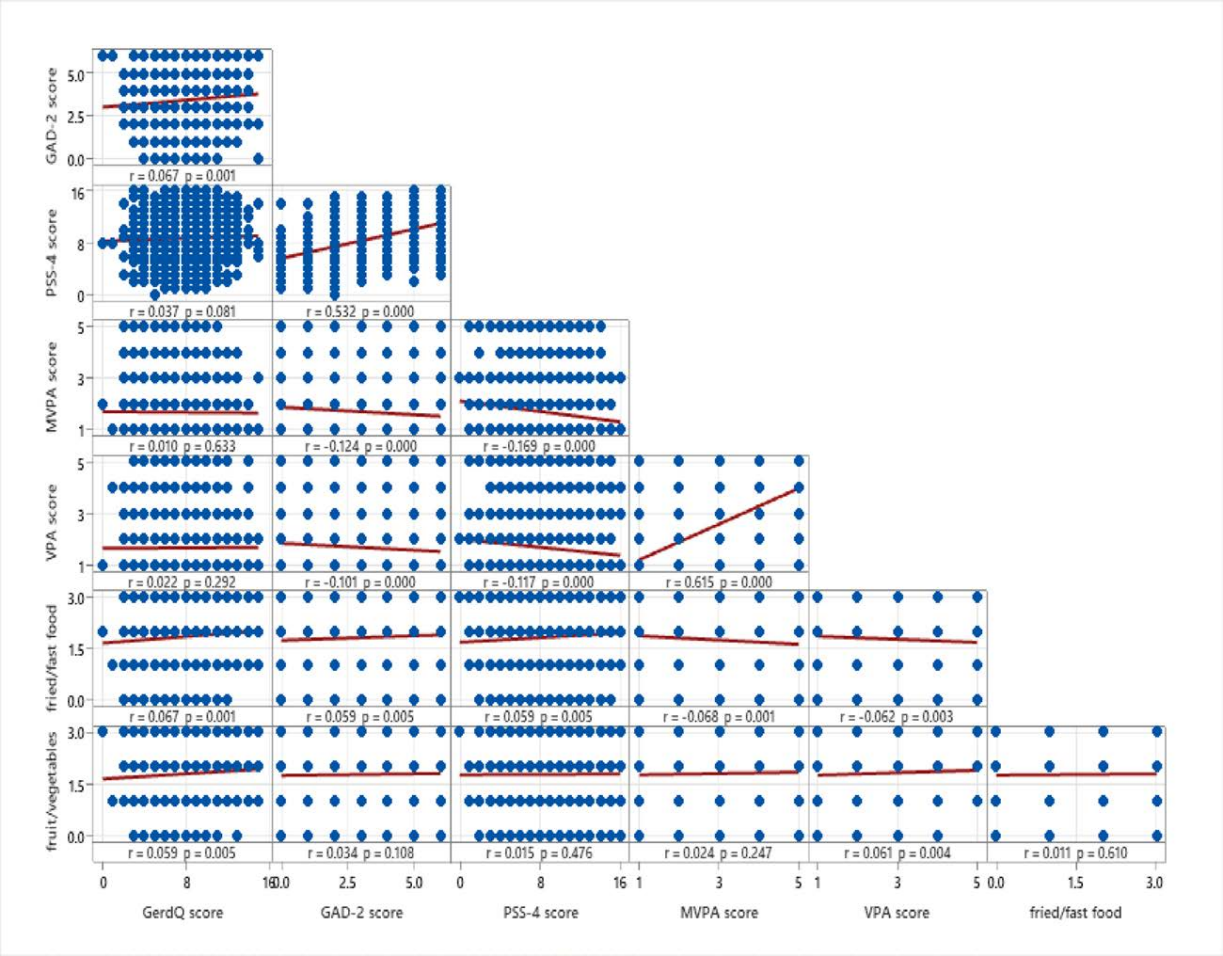
Correlation matrix showing relationships between psychological (GAD-2, PSS-4), physical activity (MVPA, VPA), GERD (GerdQ), and dietary variables (fried/fast food, fruit/vegetables). Pearson *r* and *P*-values highlight significant associations, such as stress and anxiety (PSS-4 and GAD-2), and inverse correlations between physical activity and psychological factors.

Moreover, the correlation matrix showed that there were weak yet statistically significant positive correlations observed between GAD-2 and the GerdQ score (*R* = 0.067; *P* < .05). The correlations between fried/fast food consumption and fruit/vegetable consumption with the GerdQ score were also weak (*R* = 0.067 and *R* = 0.059, respectively; *P* < .05). However, PASS-4, MVPA, and VPA scores exhibited insignificant correlations with the GerdQ score (Fig. 1).

### 3.3. The interaction effects of psychological and lifestyle factors on the diagnosis of GERD among the study participants

Individuals reporting both high stress and high anxiety exhibited a significantly higher prevalence of GERD compared to those without these factors, with a statistically significant (*P*-value < .05). Similarly, the combination of high stress and frequent fast food consumption demonstrated a significant association with GERD diagnosis (*P* < .05). Conversely, the presence of high anxiety along with frequent fast food intake did not show a statistically significant relationship with GERD (*P* > .05). In addition, factors such as low physical activity in combination with high stress or high anxiety did not exhibit significant associations with GERD diagnosis (*P* > .05) (Table 3).

**Table 3:** Interaction effects of psychological and lifestyle factors on GERD (gastroesophageal reflux disease) diagnosis.

Factors	GERD (Yes) N (%)	GERD (No) N (%)	*P*-value
High stress & high anxiety	336 (66.3%)	985 (56.8%)	≤.001*
High anxiety & frequent fast food	79 (15.6%)	218 (12.6%)	.079
High stress & frequent fast food	111 (21.9%)	297 (17.1%)	.014*
High stress & low physical activity	275 (54.2%)	924 (53.3%)	.705
High anxiety & low physical activity	215 (42.4%)	680 (39.2%)	.197
Low physical activity & frequent fast food	81 (16%)	233 (13.4%)	.147

GERD = gastroesophageal reflux disease.

* Significant.

### 3.4. Predictors of GERD among the study participants

The results of hierarchical logistic regression analysis revealed that old age, high weight, short height, and frequency of eating fried/fast food are consistently significant predictors of GERD across models. High anxiety level, as indicated by the GAD-2 score, was also estimated as a significant predictor in the final model. Conversely, medical specialty, BMI, and perceived stress are not significant predictors in this analysis (Table 4).

**Table 4:** Summary of hierarchical logistic regression analysis for variables predicting GERD (gastroesophageal reflux disease).

Independent variables Model	Model 1		Model 2		Model 3	
	Odds ratio (95% CI)	*P*-value	Odds ratio (95% CI)	*P*-value	Odds ratio (95% CI)	*P*-value
Age	1.070 (1.005–1.139)	.034*	1.079 (1.013–1.149)	.018*	1.090 (1.023–1.161)	.008*
Medical specialty	0.922 (0.729–1.166)	.497	0.955 (0.754–1.209)	.700	1.028 (0.808–1.306)	.824
Weight (kg)	1.053 (1.002–1.107)	.041*	1.058 (1.006–1.113)	.028*	1.057 (1.004–1.114)	.036*
Height (cm)	0.951 (0.910–0.994)	.027*	0.947 (0.905–0.99)	.017*	0.951 (0.908–0.996)	.035*
BMI (kg/m^2^)	0.898 (0.785–1.026)	.114	0.887 (0.774–1.016)	.084	0.890 (0.772–1.025)	.106
Frequency of eating fried food/fast food			1.194 (1.052–1.356)	.006*	1.178 (1.037–1.338)	.012*
Frequency of eating fruit and vegetables			1.180 (1.045–1.331)	.007*	1.178 (1.043–1.331)	.008*
PSS-4 score					0.996 (0.956–1.038)	.848
GAD-2 score					1.168 (1.089–1.252)	≤.001*
Chi-square	22.107		36.886		61.718	
Nagelkerke R Square	0.015		0.025		0.041	
*P*-value	≤.001*		≤.001*		≤.001*	

BMI = body mass index, CI = confidence interval, GAD-2 = The Generalized Anxiety Disorder 2-item, GERD = gastroesophageal reflux disease, PSS-4 = Perceived Stress Scale 4.

* Significant.

## 4. Discussion

GERD can manifest across all age groups, including infancy, with university students notably susceptible to it. Despite the profound impact of GERD on individuals’ daily lives, research focusing on its prevalence among higher education students in Arab nations is relatively limited despite these demographics’ heightened vulnerability to established GERD risk factors.^[23]^ This study seeks to address this gap by examining the prevalence of GERD among medical and non-medical Egyptian university students and investigating the interplay between GERD and psychological and lifestyle risk factors.

Among a cohort of 2241 university students, 22.6% had GERD diagnosis (determined by a GerdQ score exceeding 8). Various studies have consistently highlighted a higher prevalence of GERD among university students, with rates ranging from 11.8% to 52.6%.^[18,23–27]^ For instance, Baklola et al identified GERD in 17.1% of Egyptian medical students.^[18]^ In Saudi Arabia, a study by Alrashed et al revealed a 23.8% prevalence among Shaqra University students.^[23]^ In comparison, another study reported a higher rate of 33.2% among university students in the southwest region.^[27]^ The utilization of the GerdQ score as an assessment tool was common across these studies.^[23,26,27]^ University students face heightened susceptibility to GERD due to a combination of lifestyle and psychological factors that predispose them to this condition.^[27]^

Epidemiological data indicates that obesity stands out as the primary risk factor significantly associated with the occurrence of GERD, meeting multiple criteria for a causal relationship.^[24,28,29]^ In the present study, students with GERD exhibited notably higher weight and BMI compared to their counterparts without the condition, and obesity was confirmed as a relevant predictor of GERD. The emergence of GERD in obese individuals can be explained structurally as the abdominal fat’s weight raises intra-abdominal pressure and consequently heightens the likelihood of reflux episodes. Furthermore, obesity has the potential to disrupt the physiological functioning of the lower esophageal sphincter and gastroesophageal motility.^[28,30]^

University students often face numerous stressors, with a certain level of stress serving as a potential motivator for productivity and academic success. However, when stress surpasses an individual’s coping capacity, it can have adverse effects on both physical and psychological well-being.^[31]^ In this study, 86.3% of university students reported high levels of perceived stress, while 62.2% exhibited elevated anxiety levels. High anxiety levels emerged as a significant predictor of GERD, with a notable interaction effect observed in individuals reporting both high stress and high anxiety, who exhibited a considerably higher prevalence of GERD compared to their counterparts without these factors. Similarly, at Helwan University, Egypt, a significant portion of Egyptian students, around 93.2%, experienced moderate to high stress levels.^[32]^ These figures exceed the rates documented in comparable studies.^[26,33,34]^ Psychiatric conditions are frequently associated with psychoemotional disturbances that can heighten esophageal sensitivity.^[35]^ This increased sensitivity affects the brain-gut axis and alters neuroendocrine functioning, contributing to the development of GERD. Moreover, shared genetic factors between gastrointestinal and psychiatric disorders also play a significant role in GERD pathogenesis.^[36]^ Moreover, low mental health literacy and the stigma surrounding mental health, alongside the lack of time, significantly affect students’ willingness to seek help.^[37,38]^ This may explain why, despite high levels of stress and anxiety observed in the study, many students may not seek care, potentially worsening conditions such as GERD. Addressing these barriers through increased awareness and efforts to reduce mental health stigma could help alleviate psychological distress and, in turn, mitigate its physiological impacts, including GERD. In addition, the gender composition of the participants of the current study showed that 70.1% are females. This female predominance may contribute to the observed high levels of stress and anxiety. Gender differences in vulnerability to psychological conditions are proven. Females are generally more prone to experiencing these psychological burdens compared to their male counterparts.^[27,31,39]^ Psychological stress, a recognized risk factor, has been linked to exacerbating GERD symptoms through various mechanisms, including increased gastric acid secretion, delayed gastric emptying, and heightened likelihood of reflux episodes. Previous research has highlighted how stressful situations can intensify GERD symptoms, underlining the intricate relationship between psychological well-being and digestive health.^[15,40]^

Dietary choices play a pivotal role in both the occurrence and management of GERD.^[8]^ The present research has highlighted that university students who frequently consume fried and fast foods while neglecting fruits and vegetables are more susceptible to GERD. This observation aligns with a study conducted by Jarosz et al,^[41]^ which revealed a higher likelihood of reporting GERD symptoms among individuals who consume more fried and fast foods, likely due to the high-fat content in these items, which can delay gastric emptying and elevate acid reflux risks. Similarly, poor dietary patterns were linked to GERD symptoms in another study, emphasizing the impact of food habits on digestive health.^[6]^ In the Egyptian cultural context, the prevalence of fast food consumption is a rising phenomenon. For university students, the frequent consumption of fast food is often a response to time constraints, academic pressures, and social influences. Many students select quick, inexpensive, and readily available meals, which often consist of fried items, processed meats, and sugary beverages.^[42]^ This shift is particularly concerning as high-fat, fried, and processed foods are known to delay gastric emptying and increase the risk of acid reflux, both of which are significant contributors to GERD.^[43]^ On the other hand, students with a diet rich in fruits and vegetables displayed a lower prevalence of GERD symptoms. This could be attributed to the anti-inflammatory properties of such foods and their capacity to foster a healthy gut microbiome. Furthermore, diets abundant in fruits and vegetables may help reduce stomach acidity, consequently lowering the chances of acid reflux into the esophagus.^[6,44]^

A growing body of evidence indicates that maintaining high levels of physical activity can reduce the risk and prevalence of GERD.^[45]^ However, in the case of the studied Egyptian university students, despite a large proportion engaging in physical activity, no significant relationship was found between physical activity and GERD prevalence. Even when low physical activity levels were combined with high stress or anxiety, there was no significant impact on GERD diagnosis. The lack of association observed in this population may be attributed to several factors, including differences in the populations studied, variations in the methods used to assess physical activity and exercise, and differing approaches to diagnosing GERD.^[46]^ Previous research suggests that this association between physical activity and GERD is more pronounced in older adults. This could be because physical activity has a greater protective effect against the age-related decline in functional capacity in older individuals, which may not be as relevant or impactful in younger populations such as university students.^[47]^

## 5. Conclusion

In conclusion, this study underscores the intricate web of influences that contribute to the prevalence of GERD among Egyptian university students. Among the student cohort, 22.6% received a GERD diagnosis. Obesity and psychological factors were identified as pertinent predictors of GERD. Approximately 86.3% reported high levels of perceived stress, while 62.2% exhibited elevated anxiety levels. Furthermore, a significant interaction effect was noted among individuals reporting both high stress and high anxiety, who demonstrated a notably elevated prevalence of GERD. Moreover, dietary habits played a crucial role in the prevalence of GERD among university students. Those who frequently consumed fried and fast foods while overlooking fruits and vegetables were found to be more susceptible to GERD. Addressing not only the physiological aspects but also the psychological and lifestyle factors is imperative in developing holistic strategies for the prevention and management of GERD in young adults. Further research and interventions focusing on stress management, dietary modifications, and psychological well-being could prove beneficial in mitigating the burden of GERD among university students in Egypt and similar populations.

### 5.1. Strengths and limitations

The study’s strengths lie in its large sample size of 2241 participants, gathered through a stratified sampling method, ensuring a diverse representation of Egyptian university students from various regions. This inclusive sampling strategy boosts the applicability of the study’s findings to the wider university students in Egypt. Additionally, the use of validated tools like GerdQ, GAD-2, PSS-4, and NPAQ-short adds scientific rigor to the data collection process. However, the study has some limitations. The self-administered questionnaire may introduce response and recall biases. Moreover, the study’s cross-sectional design limits the ability to establish causal relationships between variables. To address this, future longitudinal studies could be valuable in establishing causality, particularly regarding the psychological and lifestyle factors involved. Such studies would allow for tracking changes over time, providing deeper insights into how these factors influence GERD development and progression.

## Acknowledgments

The research team expressed unlimited gratitude to the university students who provided us with their time and agreed to participate in our study. Moreover, we wish to express our gratitude for the valuable contributions made by Samah Shalaby Elnozahy, Shrouk Adel Farag, and Shrook Elshahat Mandour from the Faculty of Medicine at Kafr-Elsheikh University, Egypt, for their committed assistance in gathering data.

## Author contributions

**Conceptualization:** Hebatalla Abdelmaksoud Abdelmonsef Ahmed, Shady Mohamed Abdelwahab, Hoda Ali Ahmed Shiba.

**Data curation:** Hebatalla Abdelmaksoud Abdelmonsef Ahmed, Ahmed Yousef, Shady Mohamed Abdelwahab, Hoda Ali Ahmed Shiba.

**Formal analysis:** Hebatalla Abdelmaksoud Abdelmonsef Ahmed.

**Investigation:** Hebatalla Abdelmaksoud Abdelmonsef Ahmed, Hoda Ali Ahmed Shiba.

**Methodology:** Hebatalla Abdelmaksoud Abdelmonsef Ahmed, Hoda Ali Ahmed Shiba.

**Project administration:** Hebatalla Abdelmaksoud Abdelmonsef Ahmed.

**Resources:** Manal Abdulaziz Murad.

**Software:** Hebatalla Abdelmaksoud Abdelmonsef Ahmed.

**Supervision:** Hebatalla Abdelmaksoud Abdelmonsef Ahmed, Manal Abdulaziz Murad, Hoda Ali Ahmed Shiba.

**Visualization:** Hebatalla Abdelmaksoud Abdelmonsef Ahmed.

**Writing – original draft:** Hebatalla Abdelmaksoud Abdelmonsef Ahmed, Ahmed Yousef, Rania El-Kurdy, Manal Abdulaziz Murad, Shady Mohamed Abdelwahab, Hoda Ali Ahmed Shiba.

**Writing – review & editing:** Hebatalla Abdelmaksoud Abdelmonsef Ahmed, Ahmed Yousef, Rania El-Kurdy, Manal Abdulaziz Murad, Hoda Ali Ahmed Shiba.
